# Two-Photon Correlation Spectroscopy in Single Dendritic Spines Reveals Fast Actin Filament Reorganization during Activity-Dependent Growth

**DOI:** 10.1371/journal.pone.0128241

**Published:** 2015-05-28

**Authors:** Jian-Hua Chen, Yves Kellner, Marta Zagrebelsky, Matthias Grunwald, Martin Korte, Peter Jomo Walla

**Affiliations:** 1 AG Biomolecular Spectroscopy and Single-Molecule Detection, Max Planck-Institute for Biophysical Chemistry, 37077, Göttingen, Germany; 2 Division of Cellular Neurobiology, Zoological Institute, TU Braunschweig, 38106, Braunschweig, Germany; 3 AG NIND, HZI, 38124, Braunschweig, Germany; 4 Department of Biophysical Chemistry, Institute for Physical and Theoretical Chemistry, TU Braunschweig, 38106, Braunschweig, Germany; University of South Alabama, UNITED STATES

## Abstract

Two-photon fluorescence correlation spectroscopy (2P-FCS) within single dendritic spines of living hippocampal pyramidal neurons was used to resolve various subpopulations of mobile F-actin during activity-dependent structural changes such as potentiation induced spine head growth. Two major classes of mobile F-actin were discovered: very dynamic and about a hundred times less dynamic F-actin. Spine head enlargement upon application of Tetraethylammonium (TEA), a protocol previously used for the chemical induction of long-term potentiation (cLTP) strictly correlated to changes in the dynamics and filament numbers in the different actin filament fractions. Our observations suggest that spine enlargement is governed by a mechanism in which longer filaments are first cut into smaller filaments that cooperate with the second, increasingly dynamic shorter actin filament population to quickly reorganize and expand the actin cytoskeleton within the spine head. This process would allow a fast and efficient spine head enlargement using a major fraction of the actin filament population that was already present before spine head growth.

## Introduction

Changes in the shape and size of dendritic spines are closely correlated with the strength of excitatory synaptic connections and to learning and memory. It is clear that structural spine plasticity heavily depends on actin cytoskeleton dynamics [1, 2]. However, so far this could only be investigated using techniques such as fluorescence recovery after photobleaching (FRAP) which do not allow differentiating various sizes and dynamics within the mobile F-actin fraction. Thus, it is an open question how mechanistically changes in size, number and dynamics of subpopulations in the mobile F-actin fraction actually drive spine structural plasticity associated to long term potentiation (LTP). In addition, actin dynamics occur usually too fast to be resolved on a molecular level by image-based techniques. To gain a deeper insight into such fast fluctuation processes, fluorescence correlation spectroscopy (FCS) is a very valuable method [3–7]. Here we present an approach that allows simultaneously imaging of morphological changes of individual dendritic spines of living hippocampal pyramidal neurons and two-photon FCS analysis of molecular processes within their heads. FCS is based on the observation of fluorescence fluctuations caused by labelled biomolecules or other particles diffusing in and out of a very well defined, small observation volume that can be placed within cells. Analysis of these fluctuations allows a sensitive determination of the number, diffusion behavior and aggregation of different populations of the labelled biomolecules. Therefore, this approach enabled us to identify and analyze in detail the individual dynamic behavior of different mobile F-actin fractions and investigate their role during activity-dependent structural plasticity at single dendritic spines. Under basal conditions we could identify two classes of actin filaments: less dynamic and much more dynamic F-actin. Upon application of Tetraethylammonium (TEA), a protocol previously used for the chemical induction of long-term potentiation (cLTP), the FCS data suggest that longer, less dynamic actin filaments are first cut two to three times creating shorter and more dynamics fragments that cooperate with the second, increasingly dynamic short filament population to quickly reorganize and expand the spine actin cytoskeleton.

## Results

**Fig 1:**
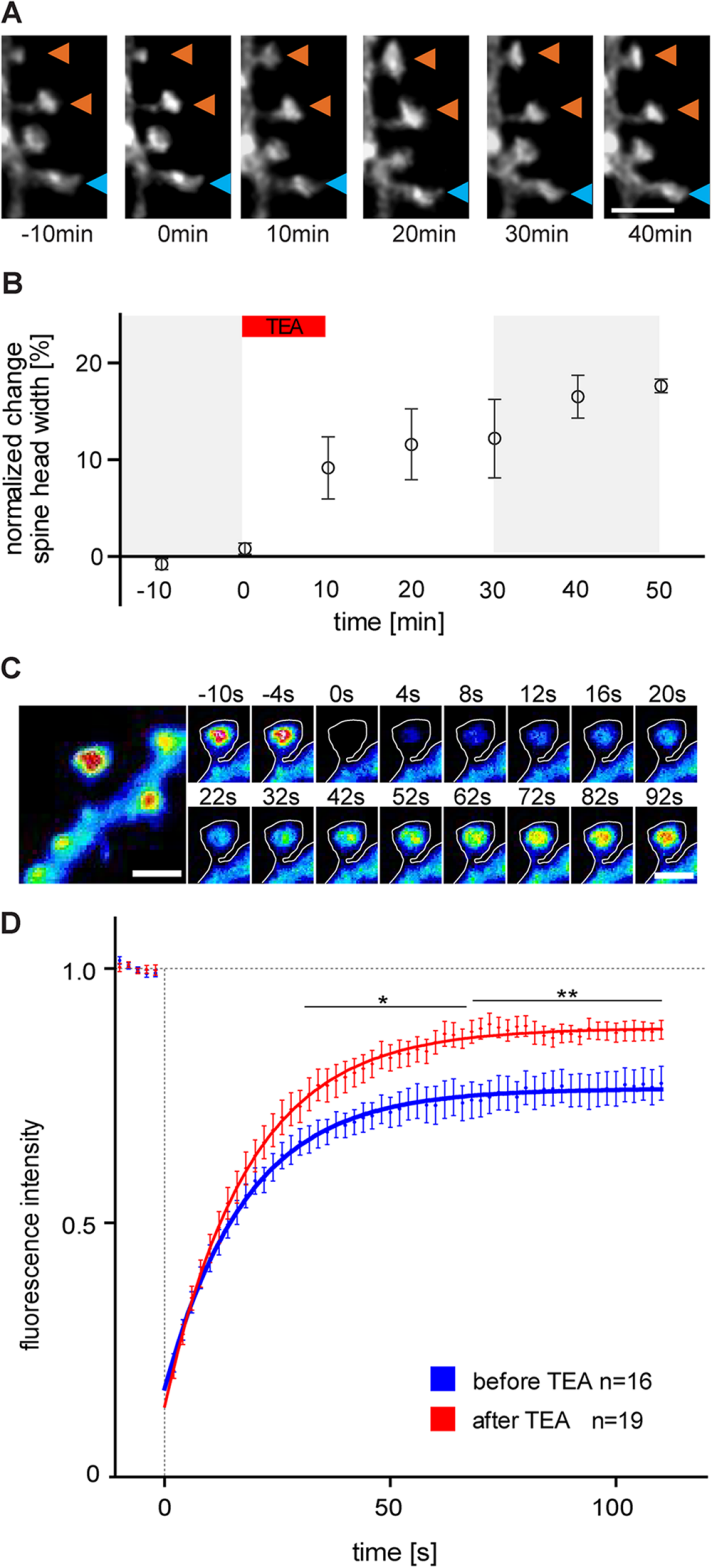
TEA application affects dendritic spine size and actin dynamics at CA3 hippocampal pyramidal neurons. **A.** Maximum projection of a stack of multiple optical sections, showing a part of the apical dendrite of a CA3 pyramidal cell expressing fCherry. Representative images of dendritic spines before (15 min) and after (30–50 min) TEA application. The orange arrowheads point to spines exhibiting a substantial increase in their head size, the blue arrowhead indicates a spine remaining stable over time upon 10min TEA treatment. Scale bar, 2μm. **B.** Quantification showing the change of spine head diameter at different time points before (15min, grey area) and after (30–50 min, grey area) 10min TEA treatment. Error bars represent SEM. (40min after TEA stimulation spine head increase of 16.52% ± 2.2%, p = 0,0012; n = 5 independent experiments / 139 spines of 5 CA3 pyramidal cells). **C.** Maximum projection before bleaching (on the left) showing the F-actin accumulation in the spine head, (pseudocolor encodes for fluorescence intensity). Scale bar, 2μm. Time series (on the right) showing the fluorescence recovery after photobleaching (FRAP) of eGFP-actin at a single spine. Time point of bleaching at 0 sec (pseudocolor encodes for fluorescence intensity). Scale bar, 2μm. **D.** Fluorescence recovery curve for eGFP-actin at single spines after photobleaching performed before (n = 16) and after (n = 19) TEA treatment (25mM TEA for 10min). The fluorescence intensity (eGFP-actin) of a single spine is blotted against the time. (plateau level at 110sec after belching before TEA 0.774 ± 0.034 *vs*. after TEA 0.88 ± 0.017; p = 0.0056).

In our study, we used TEA application in a well-established protocol previously used to chemically induce LTP both in acute hippocampal slices and cultured hippocampal neurons [8–10] and first confirmed the previously observed [10–12] increase in spine head volume by repetitively imaging a defined stretch along the apical dendrite of CA3 hippocampal pyramidal neurons before and after TEA application (Fig 1A). Upon TEA the average spine head width was significantly increased in comparison to the control observation period (in 40min up to 17%, p = 0.0012; Fig 1B). As reported previously [13, 14] not all spine head enlargements occurring upon LTP/glutamate uncaging were persistent. To determine how the actin cytoskeleton dynamics change upon TEA, FRAP was performed at individual dendritic spines of eGFP-actin expressing CA3 pyramidal cells before (15 min) and after (30–50 min) TEA application. The actin cytoskeleton of single dendritic spines was bleached and imaged at 2s intervals over 2min (Fig 1C). Starting 32s after bleaching the recovery curve recorded during TEA application showed significantly higher intensity values than under control conditions (Fig 1D). This difference was even stronger after 60s (Fig 1D). Moreover, TEA application led to a significant decrease in the stable actin filament fraction (10.2 ± 1.5% vs. 21.9 ± 3.3% before TEA, p = 0.0021) (Fig 1D and S1 Table) and a significant increase in the dynamic actin filament fraction (78.6 ± 2.2% vs. 64.2± 3.3% before TEA, p = 0.0008). The monomeric G-actin fraction and the turnover time were not altered.

**Fig 2:**
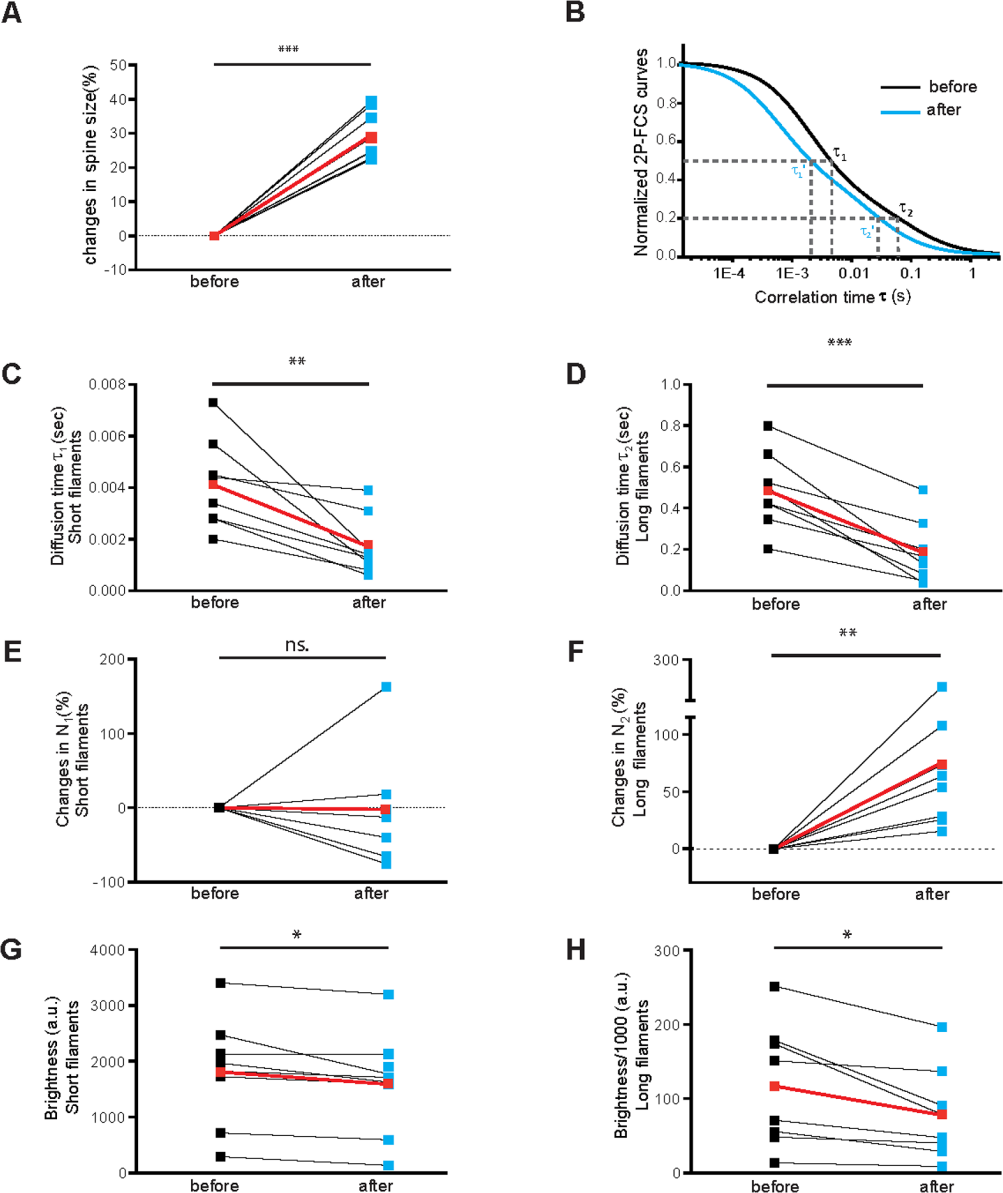
Dynamics of actin filaments within single dendritic spine with TEA treatment. **A.** Enlargement (29 ± 2%, p = 0.0009) of the sub-set of dendritic spines (n = 8) that exhibited significant growth after TEA (~ 66% of the total number of observed spines). Averages are shown in red color. **B.** 2P-FCS analysis of the mobile F-actin fraction (which comprises according to the FRAP analysis (Fig 1 and S1 Table) ~64% of the total actin) of the same dendritic spines of Fig 2A. The actin dynamics were characterized by a two component fit to the 2P-FCS curves observed before (black line) and after the morphological changes (blue line, for details see S2 Fig). From such fluorescence fluctuation analysis the diffusion times (τ_1_,τ_2_) for the very dynamic and much less dynamic filament fractions and corresponding average numbers and fluorescence brightnesses can be calculated. Please note the log τ-scale and that the curves are normalized to one for better visibility. **C.** With the treatment of TEA, the fluctuations of the more dynamic actin filaments became significant faster. The average diffusion time, τ_1_, for the same spines of A is reduced by ~59% after stimulation. Averages are shown in red color (4.1 ± 0.6 ms *vs*. 1.7 ± 0.4 ms, p = 0.0074). Black data points represent diffusion times observed within single spines before and blue data points of the same spines after TEA. **D.** The average diffusion time of the less dynamic actin filaments, τ_2_, is about 100 times larger, indicating a much higher polymerization degree than the short form. The fluctuations of the less dynamic actin filaments became also significant faster upon TEA application. The average diffusion time of the long filaments is reduced by ~64% after stimulation. Average is shown in red color (467 ± 70 ms *vs*. 176 ± 55 ms (p = 0.0008). **E.** The average, calculated number of short fragments, N_1_, in the two-photon excited observation volume did not change significantly after TEA treatment. Averages are shown in red color. (-2% ± 26.27% (p- = 0.1836)). **F.** In contrast, the average, calculated number of long fragments, N_2_, increased significantly after TEA treatment. Averages are shown in red color (75% ± 25% (p = 0.0014). **G.** The fluorescence brightness of the more dynamic fragments decreases somewhat after TEA treatment. Averages are shown in red color (1809 a.u. ± 347.2 a.u. *vs*. 1588 a.u. ± 331.5 a.u.; p = 0.0243). FCS measurements were done directly before TEA treatment and 30 minutes thereafter. **H.** The fluorescence brightness of the less dynamic fragments decreases significantly after TEA treatment. Averages are shown in red color (117200 a.u. ± 29600 a.u. *vs*. 78000 a.u. ± 22400 a.u.; p = 0.0168).

The results obtained using FRAP indicate that shortly after TEA treatment the actin cytoskeleton becomes more dynamic and thereby possibly supports the concomitant morphological changes at spine heads. However, FRAP measurements do not provide detailed information about heterogeneous filament-size populations within the mobile F-actin fraction and their dynamic behavior. Therefore, we performed 2P-FCS by placing a two-photon excited volume within various spine heads along the apical dendrite of CA3 hippocampal pyramidal and monitoring simultaneously the occurrence of morphological changes upon TEA (S1 Fig). 2P-FCS is able to differentiate various sub-groups within the mobile fraction of actin filaments that is in general only observed as a whole from the stable actin fraction in FRAP experiments. Within the well-delimited two-photon excitation volume (~200nm in diameter and ~500nm height) [15] we observed fluctuations in fluorescence occurring at different time scales and of different magnitudes caused by diffusing eGFP labeled F-actin fragments of different sizes and numbers. A statistical analysis of these fluctuations yielded fluorescence correlation curves providing detailed information such as the heterogeneity in the average times (τ) required for the different groups of filaments to diffuse through the observation volume as well as their number and brightness (see supplemental material and S2 Fig). In Fig 2 the results of this analysis are shown for the sub-set of spines (~66% of the total spine population) that displayed a significant growth upon TEA treatment (Fig 2A, average is shown in red). Grouping the mobile F-actin filaments into two different major components was sufficient to describe the correlation curves of the actin dynamics before (black) and after (blue) TEA satisfactorily (Fig 2B and S2B and S2C Fig.), even though there certainly exists a large degree of overlap in the size distributions of these two components. For one group of fragments an average diffusion time of τ_1_ ~4.1±0.6ms was observed before TEA (red dot on the left in Fig 2C) whereas the corresponding value for the other fragments was with τ_2_ ~470±70ms significantly larger (red dot on the left in Fig 2D). The diffusion time of the short fragments before treatment was about 10 times longer than the expected value for single, G-actin molecules in cells [16, 17]. Thus, these fragments likely represent short oligomers composed of ~10 actin units or G-actin bound to other proteins (e.g. actin binding molecules). According to the Rouse model [18, 19], in which the diffusion time of a polymer is approximately proportional to its monomer number, the larger fragments likely consist of ~ two orders of magnitude more monomers than the shorter fragments. Fig 2C and 2D show that the dynamics of both groups became significantly faster after TEA application, as reflected by the smaller average diffusion times τ_1_´ (1.7±0.4ms, red dot on the right in Fig 2C) and τ_2_´(180±55ms, red dot on the right in Fig 2D), respectively. In addition, the black lines for each individual spine head in Fig 2C and 2D demonstrate that the diffusion times decreased for each individual spine without any exception. The observed decreases in the average diffusion times of both forms (59% and 64%, p = 0.0074 and 0.0008, red lines in Fig 2C and 2D, respectively) indicate an average reduction in the filament sizes by factors of about 2 to 3 [18, 19]. It is unlikely that the observed change in the diffusion time by a factor of almost three can be explained by changes in the cytoplasm viscosity that is known to be similar to that of pure water. Moreover, Fig 2E and 2F indicate that upon TEA treatment induction the number of long filaments (Fig 2F) increased significantly (p = 0.0014) whereas the same number did not significantly change for the short filaments (Fig 2E). The reduction in the calculated brightness for the short and the long filaments (Fig 2G and 2H, p = 0.024 and 0.017, respectively) corroborates a reduction in size of these filaments upon TEA application. The total fluorescence intensity observed from the fixed two-photon excited volume within the spines—not to be confused with the overall fluorescence intensity of an entire spine head—did not change significantly (p = 0.131, S3D Fig). This observation indicates that on average the total concentration of labeled actin subunits, including all G-actin and all F-actin subpopulations, did not increase during TEA treatment induced spine head growth even though the total number of actin subunits seemed to be increased in the entire head.

**Fig 3:**
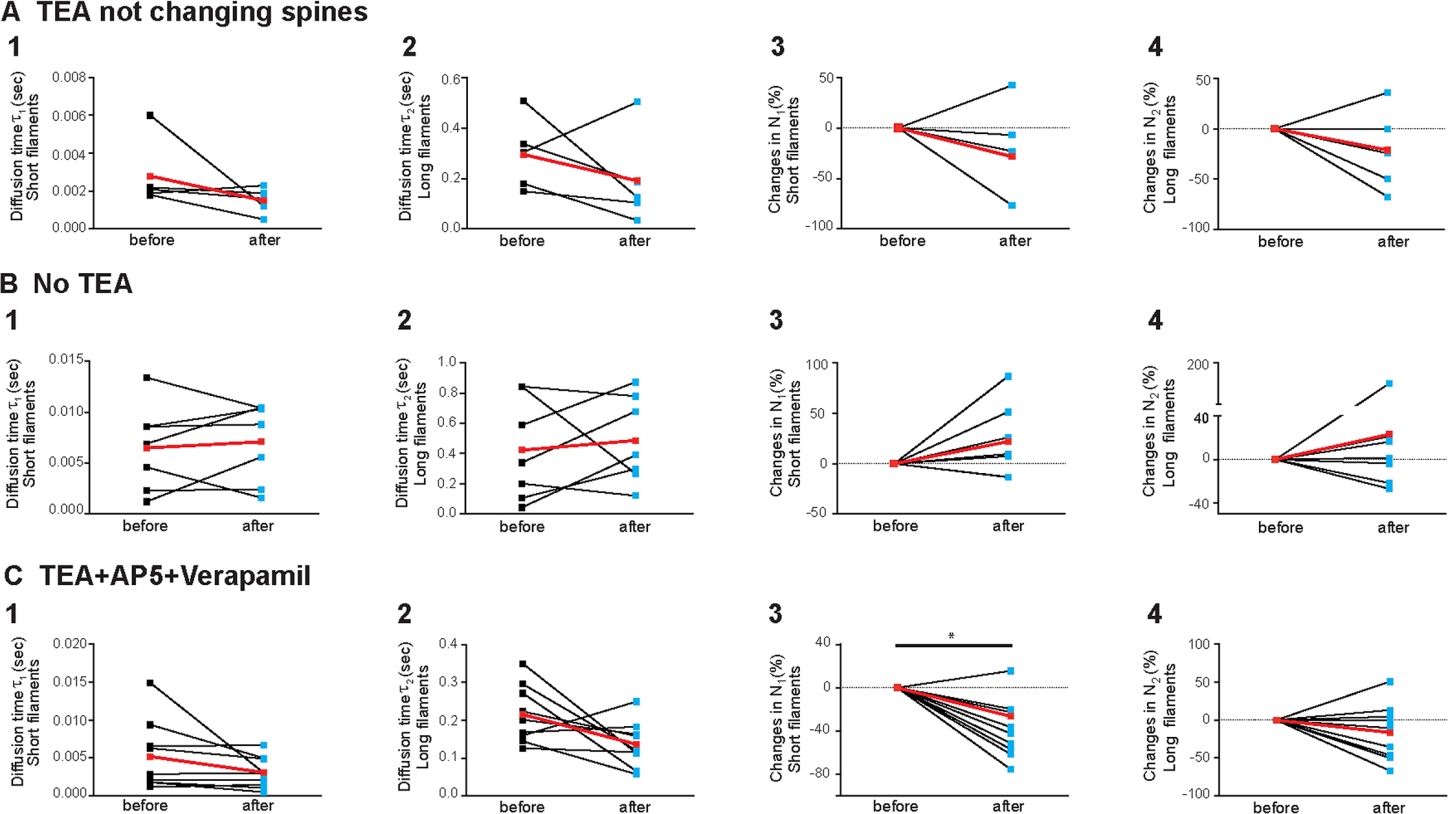
Actin dynamics within single dendritic spine under different conditions. **A.** Actin dynamics within the sub-set of dendritic spine that did not display a morphological change upon TEA treatment (n = 5). In contrast to the spines that displayed morphological changes (Fig 2) no significant changes in the diffusion time τ_1_ and τ_2_, of very dynamic (1, 2.8 ± 0.8 ms *vs*. 1.5 ± 0.3 ms, p = 0.2288) and less dynamic (2, 296 ± 64 ms *vs*. 192 ± 82 ms, p = 0.3276) actin filaments, respectively, occur. Also, no significant changes in the particle number’s (3 and 4) occur (-28.6 ± 22.5%, p = 0.31 and -21.9 ± 18.4%, p = 0.27, respectively). Averages are shown in red color. FCS measurements were done directly before TEA treatment and 30 minutes thereafter. **B.** Intrinsic actin dynamics within dendritic spines without TEA treatment. Actin filaments within living cells are highly dynamic in their equilibrium states even when the dendritic spines did not receive TEA (n = 7). Nevertheless, when no treatment is applied there is no significant change in the diffusion time, τ_1_ and τ_2_, of very dynamic (1, 6.5 ± 1.6 ms *vs*. 7.1 ± 1.5 ms, p = 0.6225) and less dynamic (2, 424 ± 127 ms *vs*. 487 ± 109 ms, p = 0.6353) actin filaments during an observation period similar as in the TEA experiments (Fig 2). Again, also no significant changes in the particle number’s (3 and 4, 21.9 ± 13.7%, p = 0.13) and 23.3 ± 26.2%, p = 0.32, respectively) occur. Averages are shown in red color. The FCS measurements were done with a time gap of 30 minutes. **C.** Co-treatment with TEA and AP5 and Verapamil. Adding AP5 and Verapamil to the TEA treatment, prevented the morphological changes at dendritic spine (n = 9). Again, no significant change occur in the diffusion time, τ_1_ and τ_2_, of very dynamic (1, 5.2 ± 1.5 ms *vs*. 3.1 ± 0.7 ms, p = 0.14) and less dynamic (2, 216 ± 25 ms *vs*. 136 ± 20 ms, p = 0.061) actin filaments, respectively, and in the particle numbers of less dynamic (4, -15.6 ± 12.4%, p = 0.1126) actin filaments. Only the number of very dynamic (3) actin filaments indicate a decrease (3, -39.0 ± 9.1%, p = 0.013) but comparing to the other filament numbers in all controls (Fig 3A3, 3A4, 3B3, 3B4, 3C3 and 3C4) this might also be a statistical outlier. At least this decrease is not nearly as significant as the increase observed in the less dynamic filament number, N_1_ (Fig 2F, p = 0.0014), and the dynamics of the less dynamic filaments, τ_2_ (Fig 2D, p = 0.0008), of those spines that exhibited significant growth after TEA. FCS measurements were done directly before the treatment and 30 minutes thereafter.

In order to prove the correlation between the changes in actin filament dynamics and the spine head growth upon TEA, we performed the same FCS analysis under three different control conditions. First, the FCS analysis was performed for the hippocampal pyramidal neurons that underwent the TEA treatment but selecting spines that did not undergo significant morphological changes (Fig 3A). In addition, we analyzed spines of neurons where no TEA treatment was applied (Fig 3B) or where TEA was applied together with the NMDA-receptor antagonist AP5 and the L-type calcium channel blocker verapamil (Fig 3C). Whereas τ_1_ and τ_2_ decreased without any exception for each individual spine that changed its morphology upon TEA treatment (Fig 2C and 2D) this is clearly not the case for the spines unaffected in their morphology (Fig 3A1 and 3A2), not treated at all (Fig 3B1 and 3B2) or upon application of AP5 and verapamil (Fig 3C1 and 3C2). In the controls individual spines showed also increasing as well as decreasing values and the average change was much smaller and not significant. Similarly, these controls showed also no significant increase in the long filament number N_2_ (Fig 3A4; Fig 3B4 and 3C4) in contrast to the consistently increasing values for growing spine population shown in Fig 2F.

In summary, all spines that displayed significant morphological changes upon TEA treatment (Fig 2A) also displayed a highly significant increase in dynamics of the short as well as the long actin filaments, τ_1_ and τ_2_ (Fig 2C and 2D, p = 0,0078 and 0,0008, respectively), and an increase in the calculated number of long filament, N_2_ (Fig 2F, p = 0,0014). On the contrary, neither spines unaffected in their morphology (Fig 3A) nor untreated (Fig 3B) nor spines treated with the NMDA-receptor antagonist AP5 and the L-type calcium channel blocker verapamil (Fig 3C) revealed significant effects (p for the same parameters τ_1_, τ_2_ and N_2_ always >0.05).

## Discussion

**Fig 4:**
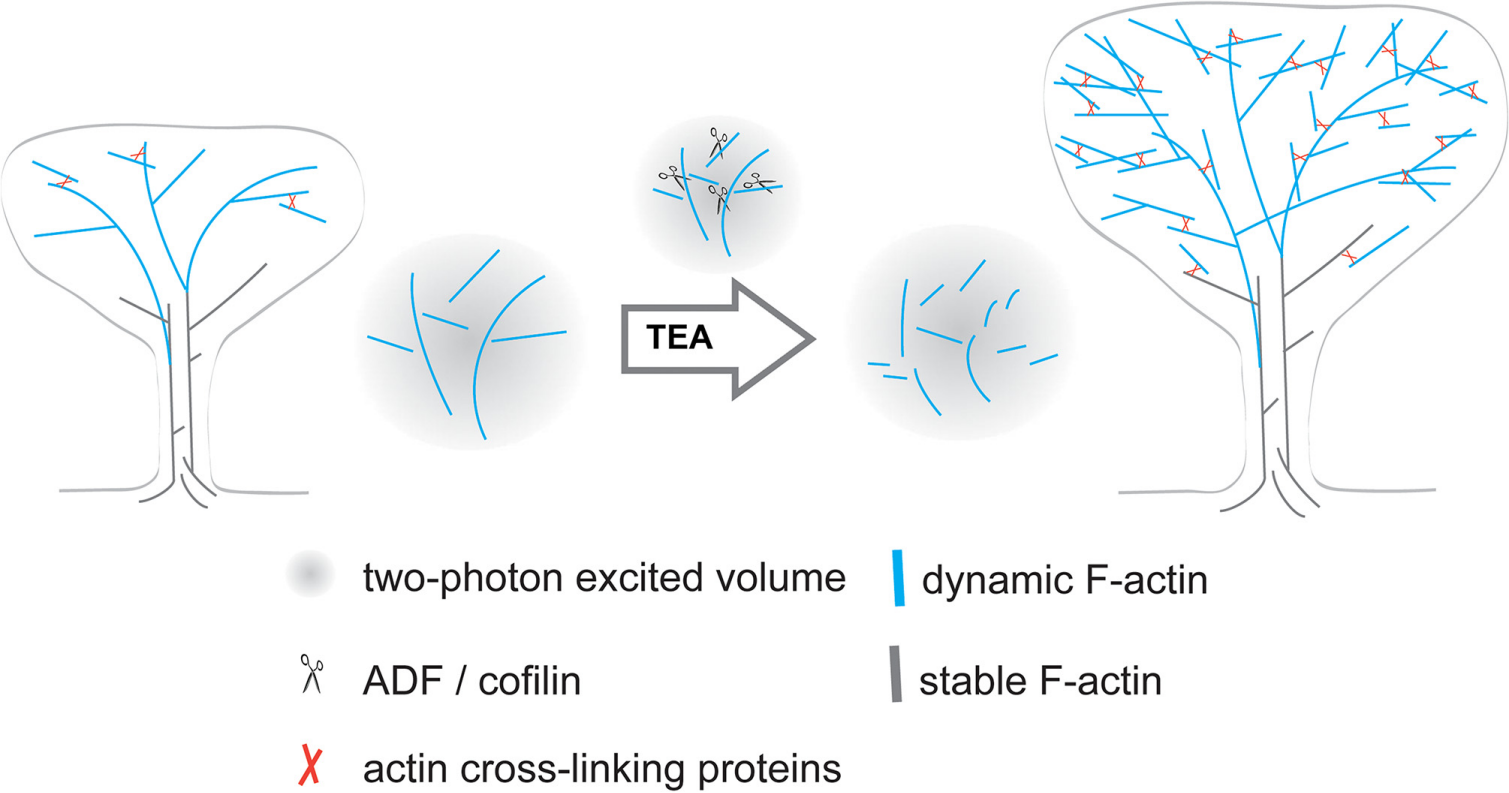
Model for the structural rearrangements actin filaments upon TEA induced spine head enlargement. Model for the structural rearrangements indicated by our observations. The gray area symbolizes the focal volume that is excited by the two-photon excitation and analyzed by FCS within the center of the spine heads.

The FRAP experiments confirmed (Fig 1) that most of the actin in spines is highly dynamic [20] (mobile fraction of ~79% composed of 65% mobile F-actin and 14% G-actin, S1 Table). The 2P-FCS analysis showed that both identified groups of mobile F-actin filaments within single spine heads, the very dynamic and much less dynamic group, became much more dynamic exclusively upon an increase in spine head volume during application of Tetraethylammonium (TEA), a protocol previously shown to chemically induce long-term potentiation. These changes indicate a reduction in filaments size of both groups which is further supported by their decreased molecular brightness. While upon spine head growth the calculated number of the less dynamic mobile filaments increased significantly within the fixed observation volume (Fig 2F), the total number of actin subunits (including all G-actin and F-actin subunits, S3A Fig) as well as the number of the much more dynamic filaments did not increase (Fig 2E). These observations suggest that during spine head growth longer filaments in the mobile F-actin fraction are cut into two to three times shorter filaments (Fig 4), albeit these newly formed “shorter” long filaments are still two orders of magnitude larger than a shorter, much more dynamic filament sub-population (compare diffusion times in Fig 2C with 2D after potentiation). We propose that cutting longer fragments by proteins such as cofilin and cross-linking them by proteins such as α-actinin provides a very fast and efficient way to reorganize the actin network upon TEA induced spine growth (Fig 4). Our observations are in agreement with a model in which newly generated shorter filaments are used to “push” the spine head to its enlarged size. This model explains unpretentiously how a spine head can be extended very efficiently during activity-dependent growth by simply using a major fraction of actin filament population that was already present in a spine head before its growth.

## Materials and Methods

### Ethics statement

The experimental protocols used in this study were carried out in conformity with the Directive 2010/63/EU of the European Parliament and the Council of the European Union and all procedures used in this study were carried out according to the guidelines approved by the Animal Committee on Ethics in the Care and Use of Laboratory Animals for the TU Braunschweig, Germany (Az §5 (02.05) TschB TU BS).

### Preparation of organotypic hippocampal slice cultures

Hippocampal organotypic cultures were prepared from postnatal day 5 (P5) C57BL/6 wild-type (WT) mice (derived from the colony at the TU Braunschweig) as previously described [21]. Neonatal mice were decapitated before the hippocampi were dissected in ice-cold sterile Gey’s balanced salt solution (GBSS) and sliced transversally at a thickness of 400μm using a McIllwain tissue chopper. The slices were placed on Millicells CM membrane inserts (Millipore) and cultivated in a 37°C, 5% CO_2_, 99% humidity environment in a medium containing 50% BME (Eagle, with Hanks salts without glutamine), 25% HBSS, 1ml of glucose (50%), 25% donor equine serum (HyClone), and 0.5 ml of L-glutamine (200 mM stock solution) for 100ml. To reduce the number of non-neuronal cells, a mixture of antimitotic drugs (cytosine arabinoside, uridine, and fluorodeoxyuridine; 10^–6^–10^–7^ M each; Sigma-Aldrich) was applied for 24h.

### Transfection

To visualize dendritic spines, hippocampal pyramidal neurons were co-transfected with expression vectors carrying either a farnesylated form of mcherry (fcherry) [22] or eGFP-actin (CA Schoenenberger, University of Basel, Switzerland) under the control of a CMV promoter. Co-expression of eGFP-actin and fcherry was confirmed by microscopy.

#### Particle-mediated gene transfer

Hippocampal slice cultures were transfected after 14–18 days in vitro (DIV) using the Helios Gene Gun System (Bio-Rad). For the co-transfection, the DNA ratio was 1:2 eGFP-actin to fcherry for a total of 2g of DNA/mg gold. The slices were transfected by shooting at a pressure of 80–100 psi and a filter, with a pore size of 3 μm was used to prevent gold clumps from damaging the slices. The slices were used for FCS measurements 12 to 24 h after transfection.

#### Single cell electroporation

Pyramidal cells in the CA3 region of hippocampal slice cultures were electroporated after 14–18 DIV using the Axoporator 800A (Axon Instruments / Molecular Devices Corp.). For the co-electroporation, a 1:1 DNA ratio eGFP-actin to fcherry was used with a total DNA concentration of 100ng/μl. The slices were kept in sterile 1x Hank's Buffered Salt Solution (HBSS) and electroporated with a stimulus of 5V, 200Hz, 1ms for 100ms. The tip resistance of the electrode (1.5/0,86mm; GC150F-10, Harvard Apparatus) was 5–7 MΩ at a pressure of 10–20mlbar controlled by a pressure gauge (GDH200, Greisinger). The slices were used for FRAP measurements 24 h after the single cell electroporation.

### Imaging

#### Time-lapse imaging and TEA treatment

Transfected CA3 hippocampal pyramidal neurons were imaged with a BX61WI FLUOVIEW 1000 (FV1000) Olympus system. Confocal image stacks of defined dendritic stretches were acquired in an interval of 10min using a 60x water objective (NA1.0), z-step of 0.5 μm and an image size of 1024x512 pixels. Hippocampal slice cultures were imaged in 35°C warm—HEPES buffered recording solution (140 mM NaCl, 5 mM KCl, 2 mM CaCl_2_, 1.5 mM MgCl_2_, 10 mM glucose and 25 mM HEPES, pH 7.4). The tetraethylammonium (TEA 25 mM, Sigma) solution (with 5 mM CaCl_2_, 0.1 mM MgCl_2_)was washed in for10 min.

#### Fluorescence Recovery After Photobleaching (FRAP)

All FRAP experiments were performed as previously described [23] between 15–19DIV at single spines of secondary or tertiary dendritic branches of CA3 hippocampal neurons. The Olympus system BX61WI FLUOVIEW 1000 (FV1000) was used to excite eGFP-actin with a 488nm. The power of the excitation laser was adjusted to a low level (~1%; 6.9 μW) to reduce photobleaching as much as possible still achieving a good signal-to-noise ratio. Scan speed was set to 8μs per pixel and a Kalman of two were used for an image size of 640 × 128 pixels with a 60x water objective (NA1.0). To increase the z-section’s depth the pinhole was opened to 400 μm. The photobleaching of a single spine was performed using the FRAP-unit FV5-LDPSU at a wavelength of 405nm and a power of 26% (∼2.3 mW) for 25 ms. To determine the baseline and the fluorescence recovery several images were taken at two second intervals before and after bleaching.

### Two photon fluorescence correlation spectroscopy (2P-FCS) combined with wide field fluorescence microscopy (FM)

For fluorescence correlation spectroscopy an ultra-fast two photon laser (900 nm, ~100 fs pulses at a repetition rate of 90 MHz) was used as light source and was combined by a dichroic mirror with a continuous mode laser (568nm) as light source for fluorescence microscopy (S1 Fig). This combination enabled the observation of morphological changes of dendritic spines by imaging of the one-photon excited membrane-fluorophores (fcherry) with a CCD and of the dynamics of the two-photon excited eGFP labeled actins through detection by an APD and subsequent 2P-FCS-analysis. The two photon focus was positioned into spines by moving the sample in the x and y direction and by moving the microscope objective in the z-direction. Great care was taken that the z-position of the two-photon focus overlaps with the focal plane of the wide-field detection. The two-photon focus was exactly placed into the center of the spine heads by first using a residual fraction of the two-photon spot visible in the CCD image and subsequent maximizing the APD-detected fluorescence observed from the two-photon excitation spot within the spine heads.

### Data analysis

Images were analyzed using the Olympus software FV1000, by drawing defined regions of interest (ROI) around the bleached spine, background and dendrite as described [23] The average intensity of the ROIs was calculated and the background fluorescence was subtracted. A bleaching correction derived from neighboring regions was also included. Fluorescent intensity from each spine was then normalized to the pre- bleach levels.

#### Calculation of the turnover time /actin pools

The turnover time (recovery half-time) is the time (sec.) necessary for the fluorescence intensity to reach half of its maximal value. The different actin fractions, stable (*f*), dynamic (*f*_*f*_) and monomeric (1−*f*_*s*_−*f*_*f*_ are derived using the equation below [2, 24]:

$$F\left(t\right)=1-f_{s}-f_{f}e^{-\frac{t}{\lambda }}$$ (1)

#### FCS-analysis

In S2A Fig the principle of FCS measurements is illustrated. Different sizes and numbers of diffusing, fluorescently labeled particles cause fluorescence fluctuations at different time scales and of different magnitudes within the small, well-delimited two-photon excited observation volume. A statistical analysis of these fluctuations using Eq 2 yield fluorescence correlation curves that provide detailed information such as the times, τ_1_ and τ_2_, that particles from two different groups require on average to diffuse through the observation volume.

$$G(\tau )=\frac{<F(t)F(t+\tau )>}{<F(t){>}^{2}}-1$$ (2)

Here, *F(t)* is the fluorescence intensity observed from the two-photon excitation volume at the time t and τ is the correlation time (not to be confused with the diffusion times τ_1_ and τ_2_). For the FCS analysis of the labeled actin filaments observed in single spines, we first selected those data that did not display movements of the entire spine head. These data can be easily identified by fluctuations and thus correlation signals that occur at times significantly larger than τ ~ 1 s. An average of data that contain only intra-spine fluctuations is exemplarily shown S1B Fig Next, one- and two component models were fitted to this data (S1B and S1C Fig, respectively). Obviously, a two-component fit is necessary (S1C Fig) since otherwise significant residuals remain (blue line S1B Fig). With the two components fitting (with the consideration of particle brightness) the diffusion time (τ_1_ and τ_2_) can be robustly determined using Eq 3.

$$G(\tau )=\frac{I^{2}\cdot N_{1}\cdot M_{1}+{\left(A\cdot I\right)}^{2}\cdot N_{2}\cdot M_{2}}{{\left(I\cdot N_{1}+A\cdot I\cdot N_{2}\right)}^{2}}$$ (3)

With $M_{1}=\frac{1}{1+\tau /\tau_{1}}\sqrt{\frac{1}{1+f(\tau /\tau_{1})}}$ and $M_{2}=\frac{1}{1+\tau /\tau_{2}}\sqrt{\frac{1}{1+f(\tau /\tau_{2})}}$

Here, *I* is the particle brightness of the short form and *A·I* the particle brightness of the long form with *A* being the factor between these two intensities. *N*_*1*_ and *N*_*2*_ are the average filament numbers in the observation volume of short and long filaments, respectively. *f* is an instrumental parameter describing the shape of the focal volume.

Based on the Rouse Model, the diffusion time of a polymer chain is roughly linearly proportional to the numbers of the monomers. Therefore, we restricted in the fitting procedure the brightness factor *A* to a range of 0–150 as it is known from the diffusion times that the two groups differ by not more than two orders of magnitude in size. Fig 2B shows the fitted functions averaged over all spines that exhibited significant growth before (black) und after (blue) TEA treatment.

#### Statistical analysis

The statistical analysis was performed using Microsoft Excel, Origin and GraphPad Prism. The FRAP analysis data were tested by applying an unpaired two-tailed Student t test point by point. The FCS analysis data were tested by applying paired two-tailed student t test. Values of p < 0.05 were considered significant. All data are presented as mean ± standard error of the mean (SEM).

## Supporting Information

S1 Fig Experimental set-up for combined two-photon fluorescence correlation spectroscopy and one-photon fluorescence microscopy. The excitation sources for two-photon fluorescence correlation spectroscopy (2P-FCS) of eGFP labeled actin filaments and one photon fluorescence microscopy (1P-FM) of the fcherry labeled spine membranes were combined by a dichroic mirror DM1. The excitation light was again reflected into the microscopy objective (O) by a special designed dichroic mirror DM2 that permits the transmission of emission light of fcherry as well as eGFP. DM3 was used to separate the emission light from fcherry and eGFP. Fluorescence filters (F) were placed in front of the detectors. A CCD is used for 1P-FM imaging of the fcherry labeled membranes and an APD is used to record the fluorescence fluctuations from the eGFP labeled actin filaments within the spine heads for 2P-FCS analysis (DM: dichroic mirror, F: filter, L: lens, O: objective lens, M: mirror, APD: avalanche photodiodes, CCD: charged couple device).
(TIFF)

S2 Fig Fluorescence correlation spectroscopy and fitting methods to quantify living cell’s correlation curve. **A**, Different sizes and mixtures of fluorescently labeled filaments cause fluorescence fluctuations on different time scales and having different relative amplitudes (exemplarily shown here as black and red fluctuations) when the two-photon excited observation volume (~200–500 nm in diameter, Fig 2 I) is placed in different spines or when the observed spines were treated differently. A statistical analysis of these fluctuations by fluorescence correlation spectroscopy (correlation curve G(τ), see Eq 2) provides detailed information such as heterogeneity in the times (τ) that filament fragments require on average to move through the observation volume. The relative amplitudes of the fluctuations contain information about the average numbers, N, of the filament fragments being in the observation volume and from this number and the total fluorescence intensity information on the per filament fragment fluorescence brightness can be calculated. **B,** Exemplary FCS raw data. Fitting a model to the raw data (black curve) that assumes only one type of homogenously sized filaments results in an unsatisfying fit (red) with systematically deviating residuals (blue). **C,** Fitting a model to the raw data (black curve) that assumes that the heterogeneous groups of filament fragments can be approximated by a two-component population (Eq 3) results in a much better fit (red) with no systematically deviating residuals (blue).
(TIFF)

S3 Fig Average intensities observed from the two-photon detection volume within spines. **A**, Average intensities observed from the two-photon detection volume within spines that exhibited significant growth after TEA before (black) and after (blue) treatment (182900 ± 69630 *vs*. 117600 ± 57850 a.u., p = 0.1310). **B**, Average intensities observed from the two-photon detection volume within spines that were not treated at all before (black) and after (blue) a time similar to the observation times in experiments with treatment (371900 ± 54980 *vs*. 443900 ± 98120 a.u., p = 0.5029). **C**, Average intensities observed from the two-photon detection volume within spines that did not exhibit significant growth after TEA before (black) and after (blue) treatment (302100 ± 86840 *vs*. 256300 ± 94660 a.u., p = 0.3030). **D**, Average intensities observed from the two-photon detection volume within spines before (black) and after (blue) treatment in which TEA treatment was combined with AP5 and Verapamil (1480000 ± 424900 *vs*. 1317000 ± 367100 a.u., p = 0.5196).
(TIFF)

S1 Table FRAP-parameter for fits shown in Fig 1D. (DOCX)
