# Supplementary figures and images for: Two-Photon Correlation Spectroscopy in Single Dendritic Spines Reveals Fast Actin Filament Reorganization during Activity-Dependent Growth
Source: PLoS One. 2015 May 28;10(5):e0128241. doi: 10.1371/journal.pone.0128241 (PMC4447372; doi:10.1371/journal.pone.0128241)

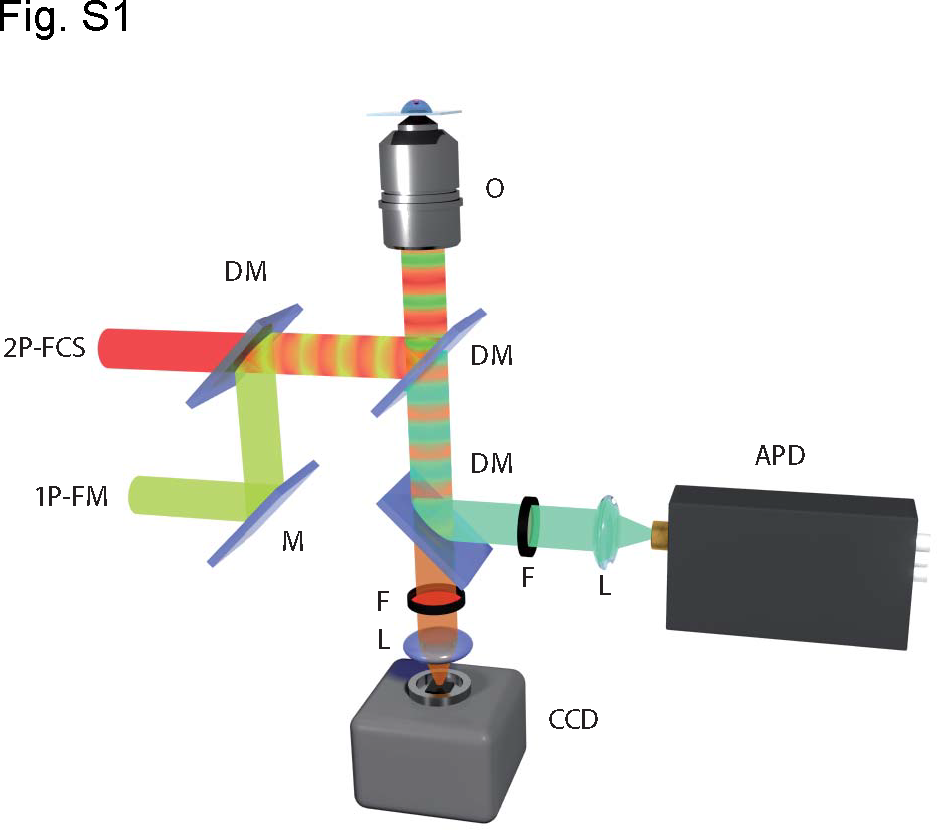

Supplement: S1 Fig — The excitation sources for two-photon fluorescence correlation spectroscopy (2P-FCS) of eGFP labeled actin filaments and one photon fluorescence microscopy (1P-FM) of the fcherry labeled spine membranes were combined by a dichroic mirror DM1. The excitation light was again reflected into the microscopy objective (O) by a special designed dichroic mirror DM2 that permits the transmission of emission light of fcherry as well as eGFP. DM3 was used to separate the emission light from fcherry and eGFP. Fluorescence filters (F) were placed in front of the detectors. A CCD is used for 1P-FM imaging of the fcherry labeled membranes and an APD is used to record the fluorescence fluctuations from the eGFP labeled actin filaments within the spine heads for 2P-FCS analysis (DM: dichroic mirror, F: filter, L: lens, O: objective lens, M: mirror, APD: avalanche photodiodes, CCD: charged couple device). (TIFF) [file pone.0128241.s001.tiff]

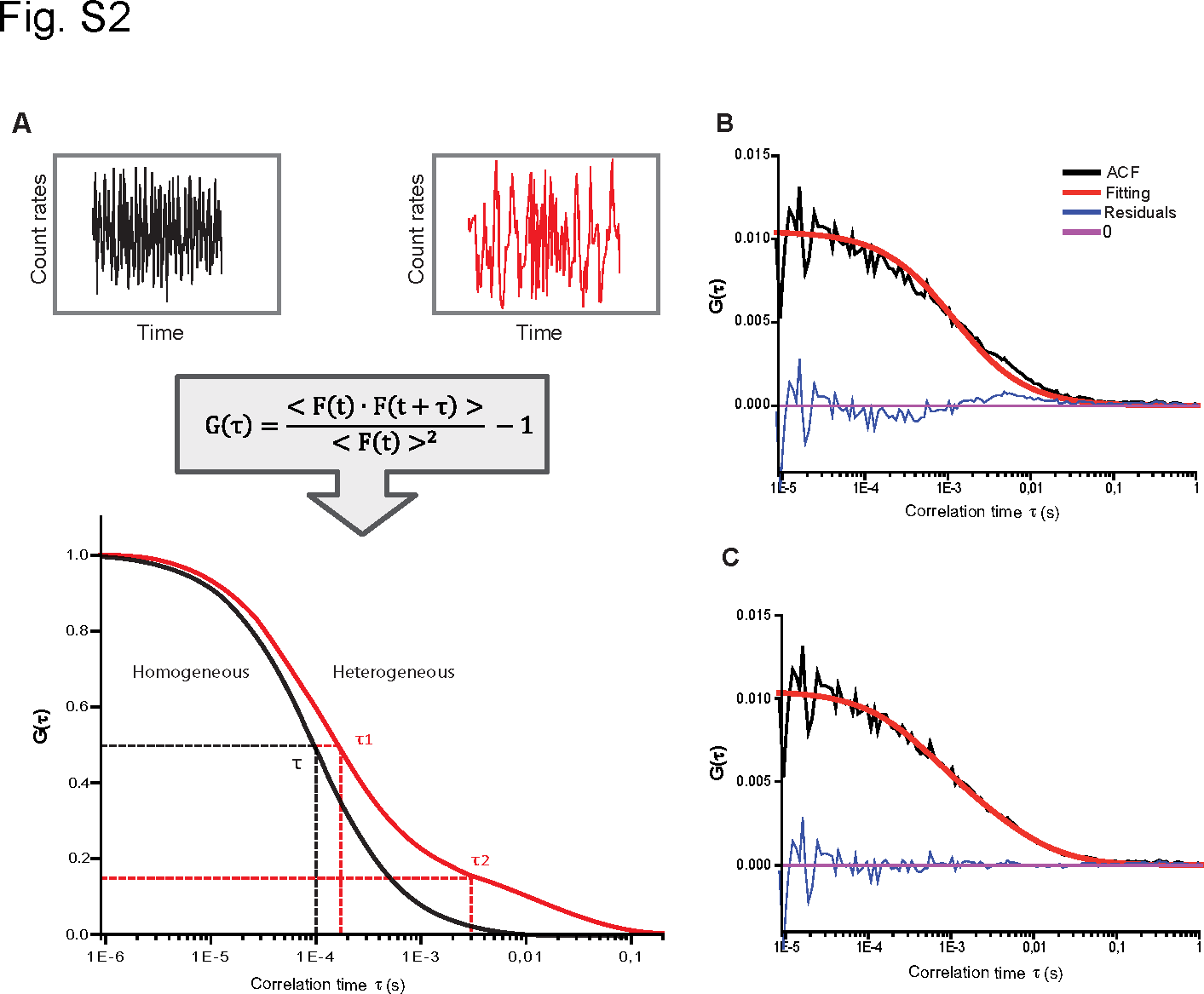

Supplement: S2 Fig — A, Different sizes and mixtures of fluorescently labeled filaments cause fluorescence fluctuations on different time scales and having different relative amplitudes (exemplarily shown here as black and red fluctuations) when the two-photon excited observation volume (~200–500 nm in diameter, Fig 2 I) is placed in different spines or when the observed spines were treated differently. A statistical analysis of these fluctuations by fluorescence correlation spectroscopy (correlation curve G(τ), see Eq 2) provides detailed information such as heterogeneity in the times (τ) that filament fragments require on average to move through the observation volume. The relative amplitudes of the fluctuations contain information about the average numbers, N, of the filament fragments being in the observation volume and from this number and the total fluorescence intensity information on the per filament fragment fluorescence brightness can be calculated. B, Exemplary FCS raw data. Fitting a model to the raw data (black curve) that assumes only one type of homogenously sized filaments results in an unsatisfying fit (red) with systematically deviating residuals (blue). C, Fitting a model to the raw data (black curve) that assumes that the heterogeneous groups of filament fragments can be approximated by a two-component population (Eq 3) results in a much better fit (red) with no systematically deviating residuals (blue). (TIFF) [file pone.0128241.s002.tiff]

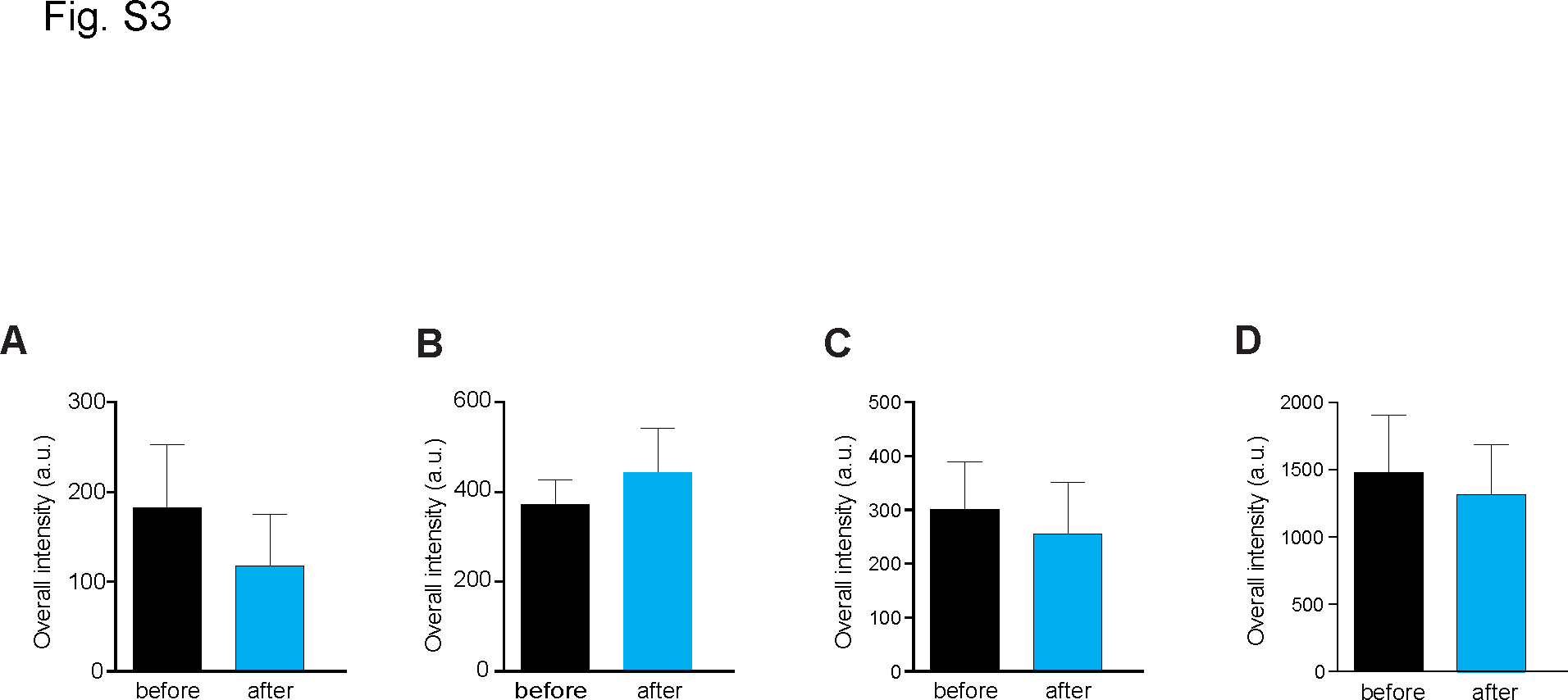

Supplement: S3 Fig — A, Average intensities observed from the two-photon detection volume within spines that exhibited significant growth after TEA before (black) and after (blue) treatment (182900 ± 69630 vs. 117600 ± 57850 a.u., p = 0.1310). B, Average intensities observed from the two-photon detection volume within spines that were not treated at all before (black) and after (blue) a time similar to the observation times in experiments with treatment (371900 ± 54980 vs. 443900 ± 98120 a.u., p = 0.5029). C, Average intensities observed from the two-photon detection volume within spines that did not exhibit significant growth after TEA before (black) and after (blue) treatment (302100 ± 86840 vs. 256300 ± 94660 a.u., p = 0.3030). D, Average intensities observed from the two-photon detection volume within spines before (black) and after (blue) treatment in which TEA treatment was combined with AP5 and Verapamil (1480000 ± 424900 vs. 1317000 ± 367100 a.u., p = 0.5196). (TIFF) [file pone.0128241.s003.tiff]
