# Supplementary material for: Two-Photon Correlation Spectroscopy in Single Dendritic Spines Reveals Fast Actin Filament Reorganization during Activity-Dependent Growth
Source: PLoS One. 2015 May 28;10(5):e0128241. doi: 10.1371/journal.pone.0128241 (PMC4447372; doi:10.1371/journal.pone.0128241)
Supplement: S1 Table — (DOCX) [file pone.0128241.s004.docx]

**Table S1**

| actin fractions: | before TEA | after TEA |
| --- | --- | --- |
| stable [%] | 21.85 ± 3.3 | 10.15 ± 1.5 |
| Dynamic [%] | 64.19 ± 3.3 | 78.56 ± 2.2 |
| G-actin [%] | 13.96 ± 2.1 | 11.29 ± 1.6 |
| Turnover time [sec] | 16.50 ± 1.8 | 14.33 ± 1.5 |
